# Metabolic Syndrome in Apparently “Healthy” Ghanaian Adults: A Systematic Review and Meta-Analysis

**DOI:** 10.1155/2017/2562374

**Published:** 2017-10-09

**Authors:** Richard Ofori-Asenso, Akosua Adom Agyeman, Amos Laar

**Affiliations:** ^1^Research Unit, Health Policy Consult, Weija, Accra, Ghana; ^2^Department of Population, Family and Reproductive Health, School of Public Health, University of Ghana, Legon, Accra, Ghana

## Abstract

**Background:**

Metabolic syndrome (MetS) is a major public health problem in Sub-Saharan Africa. We systematically reviewed the literature towards estimating the prevalence of MetS among apparently “healthy” Ghanaian adults.

**Methods:**

We searched PubMed, Web of Science, Scopus, Africa Journals Online, African Index Medicus, and Google scholar as well as the websites of the Ministry of Health and Ghana Health service through September 2016. Only studies conducted among apparently “healthy” (no established disease, e.g., diabetes and hypertension) adults aged ≥ 18 years were considered. Only studies that utilised the National Cholesterol Education Program Adult Treatment Panel (NCEP-ATP), World Health Organization (WHO), or International Diabetes Federation (IDF) classifications for MetS were included.

**Results:**

Data from nine studies involving 1,559 individuals were pooled. The prevalence of MetS based on NCEP-ATP, WHO, and IDF classifications was 12.4% (95% confidence interval [CI] = 8.3–17.4%), 6.0% (95% CI = 1.4–13.1%), and 21.2% (95% CI = 12.4–30.9), respectively. Prevalence of MetS was higher among women than men.

**Conclusion:**

Among a population of adult Ghanaians deemed “healthy,” there is a high prevalence of MetS. Preventive measures are required to address the risk components of MetS such as obesity and hypertension which are rapidly rising in Ghana.

## 1. Background

Metabolic syndrome (MetS)—a cluster of conditions—that is, increased blood pressure, high blood sugar, excess body fat around the waist, and abnormal cholesterol or triglyceride levels that occur together and increase the risk of heart disease, stroke, and diabetes, is recognised to be of significant global public health importance [1, 2]. Evidence suggests that the combined effects of the individual MetS disorders raise the risk of further disease and mortality much more than the sum of the individual risk components [3]. Subsequently, persons suffering from MetS have been found to be about two times more likely to die from and three times likely to experience heart attacks or stroke compared to persons without the condition [4]. Individuals with MetS are also about five times more likely to develop type 2 diabetes [4].

While the increased risk of type 2 diabetes and cardiovascular diseases (CVDs) consequent to MetS is widely recognised, the definition and contributions of the underlying components of MetS have been subject of debate [5]. This has sometimes been a source of confusion for clinicians regarding how MetS should be diagnosed; estimating the true prevalence of MetS in any setting is equally a challenge [5, 6]. Since 1988 when metabolic syndrome was first described [7], several definitional perspectives have emerged. The most widely used definitions/classification for MetS are those provided by World Health Organization (WHO) [8], The US National Cholesterol Education Program Adult Treatment Panel (NCEP-ATP, 2001 and 2004 revision) [9, 10], and International Diabetes Federation (IDF) [11] (Supplemental Table S1 in Supplementary Material available online at https://doi.org/10.1155/2017/2562374).

The underlying cause of MetS is not entirely understood but insulin resistance and central obesity are considered to be major players [11, 12]. Prevalence of MetS increases along with body mass index (BMI) and age. Genetics, sedentary lifestyle, a proinflammatory state, and hormonal changes may also have a causal effect, but the individual contributions of these factors may vary widely according to ethnicity [11, 13].

In spite of the challenges in the determination of MetS prevalence due to the different classifications, the IDF estimates that nearly a quarter of the world's population suffers from MetS [4]. Grundy et al. [10] reports that around 20–30% of the adult population in most countries suffer from MetS. Kaur [14] on the other hand indicates that the prevalence of MetS can vary widely across countries in the range 10–84% depending on a number of factors such as ethnicity, gender, age, and race of population under consideration.

MetS is considered to be of significant public health importance in many African countries with prevalence deemed to be on the rise and more frequent in women and urban dwellers [15]. The individual risk factors of MetS have frequently been reported among Ghanaians [16–18]. Ghana is experiencing rapid epidemiological transition which has been linked to factors such as increasing sedentary lifestyle and nutrition changes as a consequence of globalization and urbanization [19, 20]. There is concern among public health practitioners that the ongoing changes in lifestyle among Ghana's population could result in an escalation of metabolic disorders [19]. However, the burden of MetS among Ghanaian adults has not been comprehensively reviewed, although such information is critical to inform clinical practice and the design of appropriate public health preventive measures.

In this study, we sought to systematically review the available evidence of MetS among apparently “healthy” Ghanaians towards providing an estimate of the prevalence. To our knowledge, this is believed to be the first published systematic review that has attempted to estimate the national prevalence of MetS in Ghana.

## 2. Methods

This systematic review was conducted in accordance with the Preferred Reporting Items for Systematic Reviews and Meta-Analyses PRISMA guidelines [21].

### 2.1. Search Strategy

Searches for studies reporting prevalence of MetS among Ghanaians were conducted by RO in PubMed, Web of Science, Scopus, Africa Journals Online (AJOL), African Index Medicus, and Google scholar databases for entries up to 25th September 2016. The keywords used were “metabolic syndrome or dysmetabolic syndrome or syndrome X or insulin resistance syndrome or cardiovascular syndrome or cardio-metabolic syndrome” and “Ghana OR Ghanaian.” We also searched the websites of the Ministry of Health (http://www.moh.gov.gh/) and the Ghana Health Service (http://www.ghanahealthservice.org/) for nonindexed studies. The online searches were supplemented by reference screening of selected publications to identify additional studies not captured through the online searches.

### 2.2. Studies Selection

We included only studies published in English conducted on apparently healthy adults aged ≥ 18 years. Studies carried out on persons with known health disorders were excluded. Studies that mixed healthy adults and persons with known diseases were excluded unless separate prevalence was reported for the healthy population. For a study to be included, the prevalence of MetS should have been established based on an internationally recognised guideline/classification (WHO, IDF, etc., as indicated in Supplemental table S1). The quality of each study was assessed using a 12-point scoring system as adopted from the Downs and Black checklist [22]. The quality assessment focused on variables related to study objectives, characteristics of the study participants, description of inclusion/exclusion criteria, data collection methods, and appropriate data analysis techniques (e.g., whether confounders reported) and whether the discussion of the findings was appropriate and any limitations acknowledged. Individual study information and data were independently extracted by RO and AAA and crosschecked. Any discrepancies were resolved via consensus-based discussions.

### 2.3. Data Analysis

Data analysis was carried out using MetaXL and Open Meta (analyst). As adopted in similar reviews [32, 33], we conducted a meta-analysis proportion to estimate the prevalence of MetS among Ghanaians with no established disease. Individual study prevalence and pooled estimates were assessed at 95% confidence interval (CI). The level of heterogeneity was assessed based on Cohran's *Q* statistic test and degree of inconsistency (*I*^2^) [34]. As we anticipated some level of heterogeneity across studies for multiple reasons we opted to use the random effect model over the fixed effect model in the analysis of pooled effects [34]. The presence of publication bias was assessed by direct observation of funnel plots and confirmed with an egger statistic test [35]. A metaregression analysis was also carried out to assess the effects of age of participants and sampling period as possible sources of heterogeneity among the study finding. Statistical significance was set at *p* < 0.05.

## 3. Results

### 3.1. Studies Characteristics

**Figure 1:**
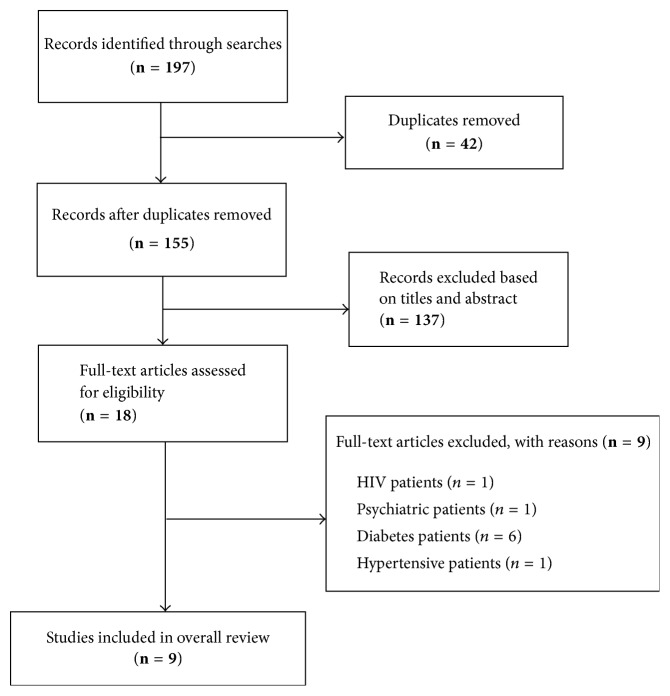
Flow chart of studies retrieval process.

Figure 1 is a summary of the steps in retrieving appropriate studies for the review. Out of 197 citations retrieved from the database searches, 18 were subjected to full text analysis following removal of duplicates and exclusion of studies based on titles and abstracts. Of the 18 studies, nine (9) met the overall inclusion criteria for addition into the review (Table 1). The nine studies excluded at this stage were mainly because they reported MetS prevalence in a group of people with known diseases (HIV, diabetes, hypertension, etc.). The 9 selected studies [23–31] were published between 2011 and 2016, although the sampling occurred during 2005–2011. The nine studies altogether included a total population of 1,559 sampled across 4 regions of Ghana. Six studies were conducted in Ashanti region [23, 25, 26, 28, 30, 31], one was conducted in Eastern region [27], one was conducted in Greater Accra [24], and one study involved participants sampled from two regions (Northern and Ashanti) [29]. Five studies had a cross-sectional design and 4 were case-control studies. The mean age of study participants across the nine studies was within the range 30.2–54.9 years. Most (89%, *n* = 8) of the studies were conducted among urban dwellers. Four studies adopted NCEP-ATP, WHO, and IDF criteria concurrently, three studies used only NCEP-ATP classification, one study used the NCEP-ATP along with the IDF classification, and another study adopted the WHO and NCEP-ATP classifications. Of the nine (9) studies that presented prevalence based on NCEP-ATP classification only six (6) studies [24, 25, 28–31] explicitly stated that the 2001 definition had been used whereas the remaining three studies did not specify if the 2001 classification or its revised version had been adopted [23, 26, 27]. In view of this, we combined all studies that used NCEP-ATP irrespective of version. Per the quality appraisal criteria, 67% of studies included in the review were graded as of high quality and 33% were graded to be of medium quality.

### 3.2. Prevalence of MetS Based on NCEP-ATP

**Figure 2:**
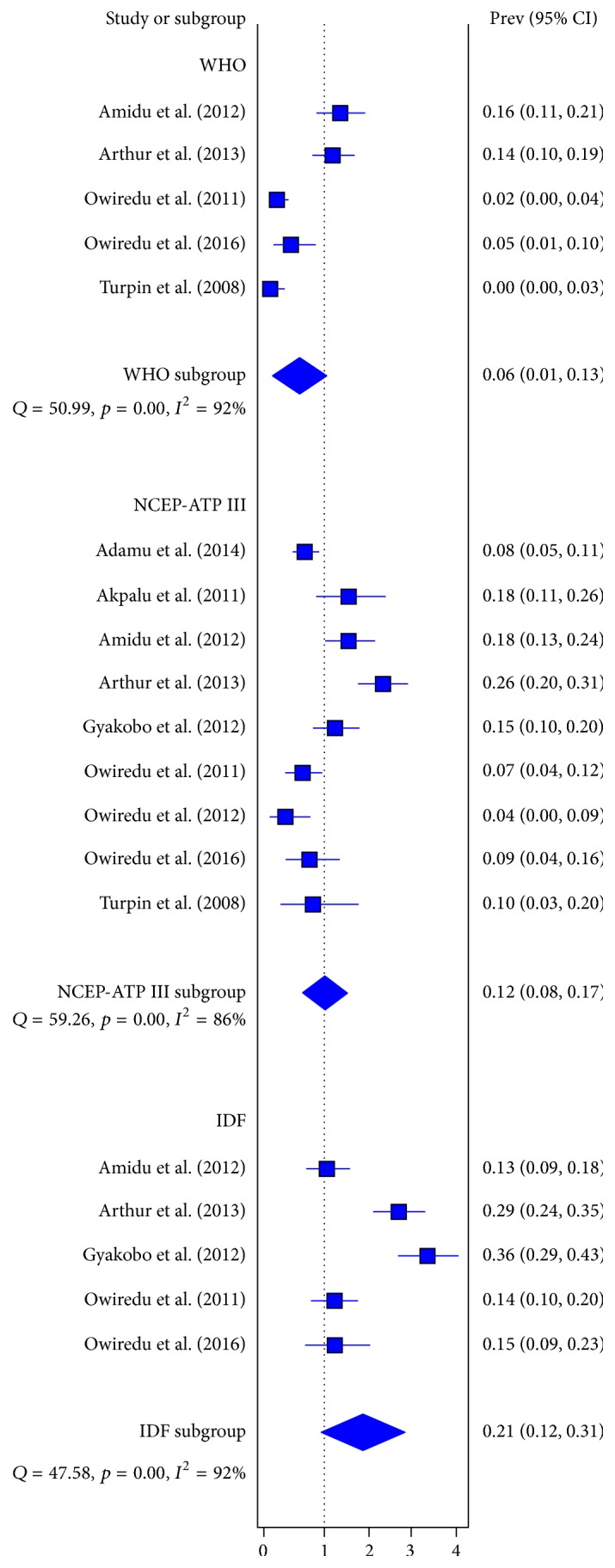
Pooled prevalence of MetS among apparently “healthy” Ghanaian adults based on different guideline classifications.

MetS prevalence based on NCEP-ATP classification was retrieved from 9 studies. The reported prevalence of MetS based on NCEP-ATP definition was within the range 3.75–25.6%. The pooled prevalence of MetS based on NCEP-ATP classification was estimated as 12.4% (95% CI = 8.3–17.4%, *I*^2^ = 0.86) (Figure 2). MetS prevalence for males based on the NCEP-ATP classification was estimated as 8.1% (3.4–14.6%, *I*^2^ = 0.87). Among females, the prevalence of MetS using the NCEP-ATP classification was 19.2% (95% CI = 12.7–26.8%, *I*^2^ = 0.82). The difference (11.2%, 95% CI = 7.7–14.7%) in prevalence of MetS among male and females based on the NCEP-ATP classification was statistically significant (*p* < 0.001).

### 3.3. Prevalence of MetS Based on IDF Criteria

The prevalence of MetS based on IDF classification was retrieved from 5 studies. The reported prevalence of MetS based on IDF definition was within the range 13.0–35.9%. The pooled prevalence of MetS among apparently healthy Ghanaian adults based on IDF classification was estimated as 21.2% (95% CI = 12.4–30.9, *I*^2^ = 0.92) (Figure 2). MetS prevalence for males based on the IDF classification was estimated as 12.3% (95% CI = 7.74–17.6%, *I*^2^ = 0.47). Among females, the prevalence of MetS using the IDF guideline was 37.2% (95% CI = 20.2–56.1%, *I*^2^ = 0.91). The gender difference (24.9%, 95% CI = 18.7–30.8%) in prevalence of MetS according to the IDF classification among male and females was statistically significant (*p* < 0.001).

### 3.4. Prevalence of MetS Based on WHO Criteria

MetS prevalence based on the WHO classification was retrieved from 5 studies. The reported prevalence of MetS based on WHO definition was within the range 0–16%. The pooled (Figure 2) prevalence of MetS based on WHO classification was estimated as 6.0% (95% CI = 1.4–13.1%, *I*^2^ = 0.92). MetS prevalence for males based on the IDF classification was estimated as 12.3% (95% CI = 7.7–17.6%). There were no statistically significant differences between MetS prevalence among males and females using the WHO criteria (*p* = 0.7768).

### 3.5. Assessment of Heterogeneity and Bias

There was no evidence of publication bias as depicted by egger regression tests which were not statistically significant, NCEP-ATP III (*p* = 0.2193), WHO (*p* = 0.1512), and IDF (*p* = 0.4111). The results of the metaregression also did not reveal any effect of mean participant age on the prevalence of MetS (*p* = 0.798). However, with each year increase in sampling period, the prevalence of MetS increased by 0.022% (coefficient: 0.022, *p* = 0.04).

## 4. Discussion

The current study aimed to systematically review the available evidence towards providing an estimate of the burden of MetS among apparently healthy adult Ghanaians. To our knowledge, this is the first systematic review that has attempted to provide an estimate of the national prevalence of MetS in Ghana.

The prevalence of MetS among Ghanaians was estimated to be in the range 6–21.2% based on three guidelines, NCEP-ATP, WHO, and IDF. The mean prevalence of MetS among adult Ghanaians is low when compared to estimates for other countries such as Iran (37.7%) [32], US (33%) [36], Australia (22.0%) [37], and Brazil (29.6%) [38] and across multiple countries in central America (30.3%) [39]. Nonetheless, comparison of the MetS prevalence across countries should be made with some caution as the condition is known to increase with age and as such countries experiencing an ageing population are likely to have higher prevalence [5]. For countries with demographic profile similar to Ghana, Oguoma et al. [40] reported a higher mean prevalence (29.2%) of MetS among Nigerians. However, their analysis included persons with established diseases like type 2 diabetes which may have contributed to the higher prevalence.

Prevalence of MetS among Ghanaians was highest with the use of the IDF classification (21.2%), followed by the NCEP-ATP guideline (12.6%) and the lowest prevalence reported with the WHO definition (6.2%). In a similar systematic review conducted among South Asians, Aryal and Wasti [41] found highest prevalence of MetS using IDF definition compared to NCEP-ATP and WHO classifications. There are at times wide discrepancies in MetS prevalence based on the guideline adopted. For instance, in the study by Turpin et al. [31], none (0%) of the participants was deemed to have MetS when the WHO definition was used, although 10% were diagnosed to have MetS per the NCEP-ATP classification. Similarly, Gyakobo et al. found an over 2-fold increase in MetS prevalence when the IDF definition was applied as opposed to the NCEP-ATP classification [27]. Obviously, the existence of multiple classifications makes precise estimation of MetS prevalence a difficult task. As expected, many researchers have called for a standardized international definition accepted by all to allow for consistency in estimation of the burden of MetS and to minimise the confusion among clinical practitioners [5, 40, 42].

The 2001 NCEP-ATP definition was revised in 2004 by lowering the thresholds for central obesity and fasting glucose as well as including patients being treated for dyslipidemia, hyperglycemia, or systemic hypertension [10]. However, the majority of studies that used the NCEP-ATP guidelines resorted to the 2001 classification. Of note a systematic review and meta-analysis by Mottillo et al. [43] that compared estimates of CVD risk associated with the metabolic syndrome using both the NCEP and revised NCEP (rNCEP) definitions in the general population and among patients without type 2 diabetes mellitus concluded that little variation exists in risk between the NCEP and rNCEP definitions of the metabolic syndrome.

Among the available classifications, it appears that the NCEP-ATP guideline is the most frequently used among Ghanaian researchers, although how this translates into clinical practice is not known. The frequent use of the NCEP-ATP guideline may be as a result of its relative simplicity. Within other countries such as Australia with advanced health systems, Cameron et al. found that the WHO definition was not practical for clinical use as compared to other definitions and suggested that the IDF definition may be better suited for CVD prevention in their country [37]. Further research will however need to focus on assessing the efficiency of the different classifications within the Ghanaian health system context.

Only one study by Gyakobo et al. was conducted among Ghanaian rural dwellers and this reported a very high prevalence of MetS [35.9% (IDF) and 15.0% (NCEP-ATP)] [27]. Earlier studies in other West African countries found much lower prevalence of MetS in rural settings [44, 45]. Although high prevalence of MetS and its associated noncommunicable diseases (NCDs) are usually blamed on Western diets, Gyakobo et al. argue that the high prevalence recorded in their study could not be easily attributed to a presumed westernization of diet and lifestyle, as these data emerged from a rural, agrarian community with traditional reliance on home-cooked foods, and a high burden of physical labour [27]. Preventive measures must therefore target both urban and rural dwellers focusing on the appropriate risk factors within these settings which are likely to vary.

As in previous reviews [32, 39, 41, 46], we observed a higher prevalence of MetS among females compared with males. Gyakobo et al. reported almost four times higher prevalence of MetS in Ghanaian females than males [27]. The authors guessed that the difference was due to the significant and higher prevalence of overweight and obesity among females (55.0%) than males (29.0%) study participants [27]. Even among women, it has been previously reported that the prevalence of MetS varies and depends on the characteristics of the population as well as the diagnostic criteria applied. Arthur et al. reported a higher prevalence of MetS among Ghanaian postmenopausal women [(43.0% IDF)] than premenopausal women [(18.9% IDF)] [26]. This observation is similar to results reported by Pandey et al. that showed higher prevalence of MetS among postmenopausal Indian women [(55% IDF)] compared to their premenopausal [(45% IDF)] counterparts [47]. Just as in other gendered health conditions, preventive efforts will need to identify sociodemographic and environmental correlates particularly those influencing women.

Prominent among suggested interventions to address MetS is lifestyle modification. A systematic review and meta-analysis published in 2012 by Yamaoka and Tango concluded that such interventions were effective in resolving MetS and reducing the severity of related abnormalities (fasting blood glucose, waist circumference, SBP and DBP, and triglycerides) in subjects with MetS [48].

Given the established association between MetS and increased risk of developing CVD [49], the findings from the current review have both clinical and public health implications. While NCDs are currently equally prevalent in developed and developing countries, their impact is far more devastating in developing countries [50]. A 2002 WHO report identified CVDs as accounting for 9.2% of total deaths in the African region in 2001, and it is estimated that by the year 2020 the number of deaths in Africa due to NCDs in general will exceed that due to communicable diseases [51]. This looming epidemic is enough reason for the early institution of appropriate interventions to reduce the risk factors for CVD, including that related to MetS [24]. In Ghana, and in other countries in the subregion, the prevalence of hypertension [16], diabetes mellitus [52], hyperlipidemia, and obesity [17, 53], which are individual components of MetS, is on the increase. To curtail the rising trend, primary prevention strategies that focus on reducing absolute CVD risk through a comprehensive risk assessment and management approach should be adopted rather than focusing on addressing isolated individual risk factors [54].

Our systematic review makes a unique contribution to knowledge regarding the burden of MetS in apparently healthy individuals. Previous studies and reviews have dwelled predominantly on persons with NCDs and other ailments. To our knowledge, this is the first systematic review and meta-analysis of studies on the subject of MetS in Ghana. The strengths of our study include the absence of publication bias and the fact that all included studies were conducted over the last decade. The MetS prevalence presented should serve as a useful starting point for understanding the burden of MetS in Ghana and for informing future research directions, design of preventive measures, and resource allocation and planning.

The following limitations of the study are worth noting. This analysis was confined to English-language articles, which could also introduce some bias as Moher et al. found language-restricted meta-analyses to overestimate treatment effect by only 2% on average compared with language-inclusive meta-analyses [21]. Considering that English is the official language of Ghana, we expect this to have a minimal effect. The studies included in this review involved participants sampled across 4 of the 10 regions of Ghana and hence the MetS prevalence estimate presented is likely to shift when more information from the other regions become available. We included only population-based studies conducted in apparently healthy adults aged ≥ 18 years; studies carried out in persons with known health disorders were excluded. A total of nine studies were excluded because they reported MetS prevalence in a group of people with known diseases (HIV, diabetes, hypertension, etc.). Given the established relationship between MetS and other conditions as diabetes and hypertension, it is possible that ours was an underestimate of the burden of MetS in Ghana.

## 5. Conclusion

Among a population of adult Ghanaians deemed to be “healthy,” there is a high prevalence of MetS. Nonetheless, the existence of multiple classifications makes precise estimation of MetS prevalence a difficult task. The efficiency of the various guidelines needs to be assessed within the Ghanaian context and efforts must be made towards defining a standard criteria for the local population. Preventive measures are required to address the risk components of MetS such as obesity and hypertension which are rapidly rising in Ghana.

## Supplementary Material

Supplemental Table SI: A summary of the most commonly used definitions and diagnosis classifications for MetS.

## Figures and Tables

**Table 1:** Descriptive characteristics of studies.

S/N	Author, year	Study design	Sampling period	Population	Mean age (years)	Sample size (*n*)	Region	Setting	Quality grade
(1)	Adamu et al., 2014 [23]	Cross-sectional	2005	Christians (population-based)	41.36 ± 13.59	387 120 (m) 267 (f)	Ashanti	Urban	Medium
(2)	Akpalu et al., 2011 [24]	Case-control	n.s	Healthy controls (no CVD)	54.9 ± 11.0	100 54 (m) 46 (f)	Greater Accra	Urban	Medium
(3)	Amidu et al., 2012 [25]	Cross-sectional	2009	Garage workers (males)	30.2 ± 7.8	200	Ashanti	Urban	High
(4)	Arthur et al., 2013 [26]	Cross-sectional	2011	Pre- + postmenopausal women	44.23 ± 0.90	250	Ashanti	Urban	High
(5)	Gyakobo et al., 2012 [27]	Cross-sectional	2007	General population	44.4 ± 6.9	206 102 (m) 104 (f)	Eastern	Rural	High
(6)	Owiredu et al., 2011 [28]	Cross-sectional	2010	Active sports individuals + sedentary persons	43.56 ± 1.06	186	Ashanti	Urban	Medium
(7)	Owiredu et al., 2012 [29]	Case-control	2007–09	Healthy matched cohorts (no CKD)	46.3 ± 1.9	80	Ashanti and Northern	Urban	High
(8)	Owiredu et al., 2016 [30]	Cross-sectional	2009-10	Healthy control (normotensive)	49.32 ± 10.10	100 55 (m) 45 (f)	Ashanti	Urban	High
(9)	Turpin et al., 2008 [31]	Case-control	2006-07	Healthy female control (no PIH)	30.22 ± 0.57	50	Ashanti	Urban	High

S/N: study number; n.s: not specified; m: male; f: female; CVD: cardiovascular disease; CKD: chronic kidney disease; PIH: pregnancy induced hypertension; WHO: World Health Organization; IDF: International Diabetes Federation; NCEP-ATP: The National Cholesterol Education Program Adult Treatment Panel.

## References

[1] Lam D. W., LeRoith D., De Groot L. J. (2000). Metabolic syndrome. *Endotext*.

[2] Misra A., Khurana L. (2009). The metabolic syndrome in South Asians: epidemiology, determinants, and prevention. *Metabolic Syndrome and Related Disorders*.

[3] Gami A. S., Witt B. J., Howard D. E. (2007). Metabolic syndrome and risk of incident cardiovascular events and death: a systematic review and meta-analysis of longitudinal studies. *Journal of the American College of Cardiology*.

[4] International Diabetes Federation (2015). *IDF Worldwide Definition of the Metabolic Syndrome*.

[5] O'Neill S., O'Driscoll L. (2015). Metabolic syndrome: a closer look at the growing epidemic and its associated pathologiess. *Obesity Reviews: An Official Journal of the International Association for the Study of Obesity*.

[6] Alberti K. G., Eckel R. H., Grundy S. M. (2009). Harmonizing the metabolic syndrome: a joint interim statement of the international diabetes federation task force on epidemiology and prevention; National heart, lung, and blood institute; American heart association; World heart federation; International atherosclerosis society; and international association for the study of obesity. *Circulation*.

[7] Reaven G. M. (1988). Role of insulin resistance in human disease. *Diabetes*.

[8] Alberti K. G. M. M., Zimmet P. Z. (1998). Definition, diagnosis and classification of diabetes mellitus and its complications. Part 1: diagnosis and classification of diabetes mellitus. Provisional report of a WHO consultation. *Diabetic Medicine*.

[9] Expert Panel on Detection Evaluation and Treatment of High Blood Cholesterol in Adults (2001). Executive summary of the third report of the National Cholesterol Education Program (NCEP) expert panel on detection, evaluation, and treatment of high blood cholesterol in adults (adult treatment panel III). *Journal of the American Medical Association*.

[10] Grundy S. M., Brewer H. B., Cleeman J. I., Smith S. C., Lenfant C. (2004). Definition of metabolic syndrome: report of the national heart, lung, and blood institute/American heart association conference on scientific issues related to definition. *Arteriosclerosis, Thrombosis, and Vascular Biology*.

[11] International Diabetes Federation (2006). *The IDF Consensus Worldwide Definition of the Metabolic Syndrome*.

[12] Carr D. B., Utzschneider K. M., Hull R. L. (2004). Intra-abdominal fat is a major determinant of the National Cholesterol Education Program Adult Treatment Panel III criteria for the metabolic syndrome. *Diabetes*.

[13] Anderson P. J., Critchley J. A. J. H., Chan J. C. N. (2001). Factor analysis of the metabolic syndrome: Obesity vs insulin resistance as the central abnormality. *International Journal of Obesity*.

[14] Kaur J. (2014). A comprehensive review on metabolic syndrome. *Cardiology Research and Practice*.

[15] Okafor C. I. (2012). The metabolic syndrome in Africa: current trends. *Indian Journal of Endocrinology and Metabolism*.

[16] Bosu W. K. (2010). Epidemic of hypertension in Ghana: a systematic review. *BMC Public Health*.

[17] Ofori-Asenso R., Agyeman A. A., Laar A., Boateng D. (2016). Overweight and obesity epidemic in Ghana - a systematic review and meta-analysis. *BMC Public Health*.

[18] Doherty M. L., Owusu-Dabo E., Kantanka O. S. A., Brawer R. O., Plumb J. D. (2014). Type 2 diabetes in a rapidly urbanizing region of Ghana, West Africa: a qualitative study of dietary preferences, knowledge and practices. *BMC Public Health*.

[19] Ofori-Asenso R., Garcia D. (2016). Cardiovascular diseases in Ghana within the context of globalization. *Cardiovascular Diagnosis and Therapy*.

[20] Agyei-Mensah S., de-Graft Aikins A. (2010). Epidemiological transition and the double burden of disease in Accra, Ghana. *Journal of Urban Health: Bulletin of the New York Academy of Medicine*.

[21] Moher D., Altman D. G., Liberati A., Tetzlaff J. (2011). PRISMA statement. *Epidemiology*.

[22] Downs S. H., Black N. (1998). The feasibility of creating a checklist for the assessment of the methodological quality both of randomised and non-randomised studies of health care interventions. *Journal of Epidemiology and Community Health*.

[23] Adamu M., Owiredu W., Plange-Rhule J. (2014). Metabolic syndrome in relation to body mass index and waist to hip ratio; a study in kumasi metropolis. *Obesity & Weight Loss Therapy*.

[24] Akpalu J., Akpalu A., Ofei F. (2011). The metabolic syndrome among patients with cardiovascular disease in Accra, Ghana. *Ghana Medical Journal*.

[25] Amidu N., Owiredu W., Mireku E., Agyemang C. (2012). Metabolic syndrome among garage workers in the automobile industry in Kumasi, Ghana. *Journal of Medical and Biomedical Sciences*.

[26] Arthur F. K., Adu-Frimpong M., Osei-Yeboah J., Mensah F. O., Owusu L. (2013). he prevalence of metabolic syndrome and its predominant components among pre-and postmenopausal Ghanaian women. *BMC Research Notes*.

[27] Gyakobo M., Amoah A. G. B., Martey-Marbell D.-A., Snow R. C. (2012). Prevalence of the metabolic syndrome in a rural population in Ghana. *BMC Endocrine Disorders*.

[28] Owiredu W., Amidu N., Gockah-Adapoe E., Ephraim R. (2011). The prevalence of metabolic syndrome among active sportsmen/sportswomen and sedentary workers in the Kumasi metropolis. *Journal of Science and Technology*.

[29] Owiredu W., Ephraim R., Eghan Jnr B., Amidu N., Laing E. (2012). Metabolic syndrome among Ghanaian patients presenting with chronic kidney disease. *Journal of Medical and Biomedical Sciences*.

[30] Owiredu W., Nkrumah C., Bedu-Addo G., Quaye L., Alidu H. (2016). Co-existence of syndrome X and hypertension among Ghanaians. *Journal of Medical and Biomedical Sciences*.

[31] Turpin C., Ahenkorah L., Owiredu W., Laing E., Amidu N. (2008). The prevalence of the metabolic syndrome among ghanaian pregnancy-induced hypertensive patients using the world health organisation and the national cholesterol education program III Criteria. *Journal of Medical Sciences*.

[32] Amirkalali B., Fakhrzadeh H., Sharifi F. (2015). Prevalence of metabolic syndrome and its components in the Iranian adult population: A systematic review and meta-analysis. *Iranian Red Crescent Medical Journal*.

[33] Li R., Li W., Lun Z. (2016). Prevalence of metabolic syndrome in mainland China: A meta-analysis of published studies. *BMC Public Health*.

[34] Higgins J. P., Thompson S. G., Deeks J. J., Altman D. G. (2003). Measuring inconsistency in meta-analyses. *British Medical Journal*.

[35] Egger M., Smith G. D., Schneider M., Minder C. (1997). Bias in meta-analysis detected by a simple, graphical test. *British Medical Journal*.

[36] Aguilar M., Bhuket T., Torres S., Liu B., Wong R. J. (2015). Prevalence of the metabolic syndrome in the United States, 2003-2012. *Jama*.

[37] Cameron A. J., Magliano D. J., Zimmet P. Z., Welborn T., Shaw J. E. (2007). The metabolic syndrome in Australia: prevalence using four definitions. *Diabetes Research and Clinical Practice*.

[38] de Carvalho Vidigal F., Bressan J., Babio N., Salas-Salvado J. (2013). Prevalence of metabolic syndrome in Brazilian adults: a systematic review. *BMC Public Health*.

[39] Wong-McClure R. A., Gregg E. W., Barceló A., Lee K. (2015). Prevalence of metabolic syndrome in Central America: a cross-sectional population-based study. *Pan American journal of public health*.

[40] Oguoma V. M., Nwose E. U., Richards R. S. (2015). Prevalence of cardio-metabolic syndrome in Nigeria: a systematic review. *Public Health*.

[41] Aryal N., Wasti S. P. (2016). The prevalence of metabolic syndrome in South Asia: A systematic review. *International Journal of Diabetes in Developing Countries*.

[42] Patel A., Huang K.-C., Janus E. D. (2006). Is a single definition of the metabolic syndrome appropriate? - A comparative study of the USA and Asia. *Atherosclerosis*.

[43] Mottillo S., Filion K. B., Genest J. (2010). The metabolic syndrome and cardiovascular risk: a systematic review and meta-analysis. *Journal of the American College of Cardiology*.

[44] Oladapo O. O., Salako L., Sodiq O., Shoyinka K., Adedapo K., Falase A. O. (2010). A prevalence of cardiometabolic risk factors among a rural Yoruba south-western Nigerian population: a population-based survey. *Cardiovascular Journal of Africa*.

[45] Fezeu L., Balkau B., Kengne A.-P., Sobngwi E., Mbanya J.-C. (2007). Metabolic syndrome in a sub-Saharan African setting: Central obesity may be the key determinant. *Atherosclerosis*.

[46] Mabry R. M., Reeves M. M., Eakin E. G., Owen N. (2010). Gender differences in prevalence of the metabolic syndrome in Gulf Cooperation Council Countries: a systematic review. *Diabetic Medicine : A Journal of the British Diabetic Association*.

[47] Pandey S., Srinivas M., Agashe S. (2010). Menopause and metabolic syndrome: a study of 498 urban women from western India. *Journal of Mid-Life Health*.

[48] Yamaoka K., Tango T. (2012). Effects of lifestyle modification on metabolic syndrome: a systematic review and meta-analysis. *BMC Medicine*.

[49] Isomaa B., Almgren P., Tuomi T. (2001). Cardiovascular morbidity and mortality associated with the metabolic syndrome. *Diabetes Care*.

[50] Reddy K. S., Yusuf S. (1998). Emerging epidemic of cardiovascular disease in developing countries. *Circulation*.

[51] World Health Organization (2002). *World Health Report;2002; Reducing Risk, Promoting Healthy Life*.

[52] Aikins A. D., Owusu-Dabo E., Agyemang C. (2013). Diabetes in Ghana: a review of research on prevalence, experiences and healthcare. *Chronic Non Communicable Diseases in Ghana: Multidisciplinary Perspectives*.

[53] Abubakari A. R., Lauder W., Agyemang C., Jones M., Kirk A., Bhopal R. S. (2008). Prevalence and time trends in obesity among adult West African populations: a meta-analysis. *Obesity Reviews*.

[54] Bovet P. (2002). Editorial: The cardiovascular disease epidemic: Global, regional, local. *Tropical Medicine and International Health*.

