# Supplementary material for: Metabolic Syndrome in Apparently “Healthy” Ghanaian Adults: A Systematic Review and Meta-Analysis
Source: Int J Chronic Dis. 2017 Oct 9;2017:2562374. doi: 10.1155/2017/2562374 (PMC5654269; doi:10.1155/2017/2562374)
Supplement: Supplementary file 1 — Supplemental Table SI: A summary of the most commonly used definitions and diagnosis classifications for MetS. [file 2562374.f1.docx]

**Supplemental Table S1:** A summary of the most commonly used definitions and diagnosis classifications for MetS

| WHO (1999) | NCEP-ATP III (2001) | IDF (2005) |
| --- | --- | --- |
| low insulin sensitivity, + any two of the below listed features;   1. Obesity   WHR > 0.90 (Male)  WHR > 0.85 (Female) and or BMI > 30 kg/m2   1. Triglycerides ≥150 (1.7 mmol/L)   Cholesterol – HDL   - <35mg/dl (Male) - <39mg/dl (Female) - Male: ≤40 (1.03 mmol/L) - Female ≤50 (1.29mmol/L)  1. BP ≥ 140/90 mmHg 2. Microalbuminuria >30 mg/g | Any three of the following;   1. WC > 102 cm (Male)   WC > 88 cm (Female)   1. Triglycerides ≥150 (1.7 mmol/L)   Cholesterol – HDL   - Male: ≤40 (1.03 mmol/L) - Female ≤50 (1.29mmol/L)  1. BP ≥ 140/90 mmHg 2. Fasting plasma glucose ≥110 mg/L or T2DM | Central obesity =WC (ethnicity and gender specific) + any two of the following;   1. Triglycerides ≥150 (1.7 mmol/L)   Cholesterol – HDL   - Male: ≤40 (1.03 mmol/L) - Female ≤50 (1.29mmol/L)  1. BP ≥ 130/ 85mmHg 2. Fasting plasma glucose ≥5.6 mmol/L or T2DM |
